# Implicating neuroinflammation in hippocampus, prefrontal cortex and amygdala with cognitive deficit: a narrative review

**DOI:** 10.1007/s13205-025-04468-2

**Published:** 2025-08-30

**Authors:** Vandana Blossom, Sheetal D. Ullal, Melisha M. D’Souza, Anu V. Ranade, Nayanatara A. Kumar, Rajalakshmi Rai

**Affiliations:** 1 Department of Anatomy, Kasturba Medical College Mangalore, Manipal Academy of Higher Education, Manipal, India; 2 Department of Pharmacology, Kasturba Medical College Mangalore, Manipal Academy of Higher Education, Manipal, India; 3 Department of Basic Medical Sciences, College of Medicine, University of Sharjah, Sharjah, UAE; 4 Department of Physiology, Kasturba Medical College Mangalore, Manipal Academy of Higher Education, Manipal, India

**Keywords:** Neuroinflammation, Hippocampus, Amygdala, Prefrontal cortex, Microglia, Astrocyte

## Abstract

Neuroinflammation is known to be a contributing factor for several neurological disorders as well as cognitive dysfunction. Different signalling pathways, and a variety of supporting cells of CNS are suggested to be involved in the progression of neurodegeneration. Among the factors contributing to neuroinflammation, peripheral inflammation takes a lead role according to recent research, since persistent peripheral inflammation is believed to disrupt the blood–brain barrier (BBB). This, in turn, allows the peripheral immune cells to infiltrate the central nervous system (CNS), triggering a chronic inflammatory response. Microglia and astrocytes, the key glial cells in the CNS, become overactivated, resulting in the unwarranted generation of the proinflammatory cytokines, such as TNF- α, IL- 1β, and the IL-6. While acute neuroinflammation is initially beneficial in repairing neuronal damage, prolonged activation contributes to the oxidative stress, mitochondrial dysfunction, protein aggregation and neural degeneration. The dysregulation of the neuroinflammatory process is likened to the deposition of the amyloid precursor proteins (APP), tau pathology and the synaptic dysfunction, ultimately impairing cognitive function. Key brain regions like the hippocampus, prefrontal cortex and amygdala are particularly vulnerable to neuroinflammatory damage. Chronic inflammation in these areas disrupts synaptic plasticity, neurogenesis and neurotransmitter stability, leading to cognitive decline and several neurological disorders. Understanding the regional specificity of neuroinflammatory responses provides valuable insights into mechanisms underlining cognitive impairment. Multifaceted treatment approaches like improvement in the delivery of drugs across the BBB, disease-specific cytokine centred treatment and improving the gut microbial environment with lifestyle changes would help in inhibiting the progression of neuroinflammation and associated cognitive dysfunction in various neurodegenerative diseases. This review is an attempt to differentiate the impact of neuroinflammation on major regions of the brain associated with cognition, so that future studies targeting neurotherapeutic strategies might get benefited, by understanding the mechanism of the inflammatory pathway that affects the brain and a spectrum of cognition. Here, we also discuss the influence.

## Introduction

Neuroinflammation, characterized by the activation of glial cells and a subsequent rise in the pro-inflammatory cytokines plays a. The regions of the brain associated with cognition, like hippocampus, prefrontal cortex, and amygdala, exhibit varied susceptibility to neuroinflammatory processes, which significantly impacts memory, learning, and emotional regulation. Central nervous system suffers neuronal cell loss and degeneration triggered by various anatomical, biochemical, or functional factors (Jo et al 2023). If neuroinflammation persists for a prolonged duration it might result in progressive or chronic neurodegeneration and subsequent neurocognitive deficits. More common, known risk factors leading to neurodegenerative diseases include advancing age, stressful conditions like infections or peripheral organ injuries and peripheral inflammation is the major pathological event leading to neurodegeneration under the above circumstances. When there is tissue injury, a protective mechanism sets in initially to heal and repair the injured tissue, which is referred as inflammation (Takeuchi and Akira 2010). Persistent or recurring peripheral inflammation may lead to neuroinflammation in the brain through several mechanisms and a damaged blood–brain-barrier (BBB) is one of the major causative factors among them. This was witnessed in long Covid patients, wherein prolonged systemic inflammation and BBB disruption were the key features leading to brain fog and associated cognitive impairment (Greene et al 2024). Table 1 describes various factors causing BBB disruption, triggering neurological disorders. Through the disrupted BBB peripheral immune cells enter the brain and initiate inflammatory process in the central nervous system, which, in excess, can further worsen the BBB destruction (Galea 2021).

**Table 1:** different factors leading to BBB dysfunction in various neurological disorders

Neurological disorder	Triggers for BBB disruption	Features	References
Multiple Sclerosis (MS)	T-cell and B-cell infiltration, cytokines, MMP activation	Chronic autoimmunity, demyelination, BBB breakdown precedes lesion formation	Alvarez et al 2015
Alzheimer’s Disease (AD)	Aβ-induced inflammation, oxidative stress, pericyte dysfunction	Vascular amyloidosis, tight junction degradation, BBB disruption	Sweeney et al 2018
Parkinson’s Disease (PD)	Systemic LPS, α-synuclein toxicity, microglial activation	Substantia nigra-specific BBB leakage, gut-brain axis, neuroinflammation	Calabresi et al 2023
Stroke (Ischemic)	Acute ROS/RNS, cytokine surge, endothelial injury	Edema, hemorrhagic transformation, rapid BBB opening	Okada et al 2020
Sepsis-Associated Encephalopathy	Cytokine storm, endothelial dysfunction, microvascular injury	BBB leak without direct CNS infection, delirium, long-term cognitive deficits	Huang et al 2021
Epilepsy	Inflammatory cytokines alter tight junctions, BBB opening precedes seizure onset	Albumin extravasation activates astrocytes, long-term barrier dysfunction	Vezzani et al 2022
COVID-19-Associated Neurological Disorders	Cytokine surge, ACE2 receptor-mediated endothelialitis, thromboinflammation	Microvascular damage, BBB permeability linked to systemic hyperinflammation	Achar and Ghosh, (2020

### Peripheral inflammation-induced neuroinflammation

The extent of peripheral inflammation induced neuroinflammation, and its impact varies among individuals, which can be attributed to genetic factors, age, gut microbiota, overall health and nature of trigger (Zhang et al 2023a). Systemic inflammation caused by gastrointestinal infections, rheumatoid arthritis, or other infections instigates chronic neuroinflammation through various mechanisms (Sun et al 2022). These mechanisms include disrupted BBB permeability, microglial activation, peripheral immune cell infiltration, gut microbiota dysbiosis. Transcription factor variations. Peripheral inflammation caused by joint pain, like rheumatoid arthritis, is associated with modulation in the cytokine IL1 system (Lampa et al 2012; Schrepf et al 2018). Neuroinflammation induced by genetic factors like variants of the APOE protein in microglia may trigger amyloid protein aggregation in some vulnerable people (Tweedie et al 2012). In-depth understanding of all these contributing factors that vary in different individuals might help in the development of targeted treatments and their efficacy.

Various therapeutic agents that target peripheral inflammation to resolve neuroinflammation are in the experimental phase (Walker et al 2023). The initial symptoms of neurodegenerative diseases, which appear following chronic peripheral inflammation include impaired memory and motor functions associated with cognitive decline, sleep disturbance, etc. Early interventions, like immune modulatory therapy, anti-inflammatory drugs, life-style interventions, like exercises, cognitive training activities, nutrient-rich diet and meditation, help to delay or revert the progression of neurodegenerative diseases, like AD, though in-depth research is needed to further strengthen it. The major cells which contribute to the progression of the inflammatory process in the CNS are microglia, astrocytes and peripheral immune cells that seep through the ruptured BBB (Di Sabato et al. 2016). Microglia and astrocytes are said to be essential for the homeostasis of normal brain physiology and whenever there is a brain insult these glial cells along with endothelia cells and perivascular macrophages help in accelerating immune response and signalling pathways (Jo et al. 2023). Furthermore, activated microglia in response to neuronal injury eventually stimulate the astrocytes also to carry on the defensive response and improves tissue repair (Lecca et al 2022). Persistent neuroinflammation sets in when the body’s natural defence mechanisms are compromised along with excessive release of proinflammatory mediators by overactivated glial cells, and this is responsible for the progression of neurodegenerative changes. This persistent inflammation of the CNS involving different immune cells and inflammatory mediators is referred to as chronic neuroinflammation. Chronic neuroinflammation significantly impacts several regions of the brain inadvertently affecting it, structurally and functionally, culminating in neurodegenerative disorders. The objective of this review article is to focus on the factors facilitating chronic neuroinflammation, its implications on various regions of the brain, especially the regions linked to cognitive functions. Various databases and search engines like PubMed, Springer, Wiley, Scopus and Web of Science were referred for collecting the data.

## Mechanism of neuroinflammation (Table 2)

**Table 2:** Depicts the mechanism of neuroinflammatory process

Mechanism	Description
Activation of Microglial cells	Microglia are triggered and activated mainly through inflammatory signals. When activated they undergo morphological alterations and attain amoeboid shape. An array of factors could lead to their activation such as infections, injury and metabolic disturbances like diabetes or by stimuli such as lipopolysaccharide (LPS) & amyloid beta (Aβ). (Maguire et al 2022; Isik et al 2023) Microglia is also involved in the release of neurotransmitters like glutamate, which inhibit MHC class II expression on microglia, further modulating their activation state. In the hilar part of hippocampus glutamate is the major excitatory neurotransmitter released by the mossy fibers (Barger et al 2007; Haroon et al 2017)
Release of Proinflammatory Factors	Activated microglia produces pro-inflammatory cytokines like interleukin-1b (IL-1b), tumor necrosis factor alpha (TNF-a), and interleukin-6 (IL-6). Elevated cytokines are generally associated with various neuroinflammatory conditions and neurodegenerative diseases (Smith et al 2012)
Inhibition of MHC class II Expression	Excessive neurotrophins can suppress the release of major histocompatibility complex (MHC) class II molecules on microglia, reducing their ability to present antigens and activate T cells. This suppression is mediated through the p75 neurotrophin receptor (Neumann et al 1998)
Infiltration of Peripheral Immune Cells	In more severe cases of neuroinflammation, peripheral immune cells like T cells and macrophages can infiltrate the CNS, further amplifying the inf lammatory response (Zhang et al 2023a, b)
CD200-CD200R Interaction	Immune homeostasis in the brain is dependent upon the interaction between CD200 expressed in the neurons and its receptor CD200R present in microglia. The significance of this connection in controlling the activation state of microglia was demonstrated in IL-4-deficient mice wherein decreased CD200 expression caused greater neuroinflammation in response to LPS (Lyons et al 2009)
Blood–Brain Barrier (BBB) dysfunction	BBB which physiologically act like a barrier against the infiltration of immune cells and other molecules, can get compromised during neuroinflammation allowing immune cells and inflammatory mediators to enter the brain, further deteriorating the pathological condition (Takata et al 2021)
Disruption of homeostasis and overall balance	The balance between pro-inflammatory cytokines (such as IL-1b, IL-6, TNF-a) and anti-inflammatory cytokines (such as IL-4, IL-10) is crucial in maintaining healthy microenvironment for neuronal function. Dysregulation of this balance, as in aging or neurodegenerative diseases, can lead to chronic neuroinflammation and neuronal dysfunction. In conditions like T2DM, neuroinflammation in the hypothalamus can disrupt the balance between orexigenic and anorexigenic neurons, leading to metabolic dysregulation. (Bhol et al 2024; Al-Sayyar et al 2023)
Role of IL-4	Interleukin-4 (IL-4) has been identified as a key regulator of neuroinflammation. IL-4 has anti-inflammatory properties and can modulate microglial activation. It can decrease microglial activation induced by LPS or Aβ protein, leading to a reduction in pro-inflammatory cytokine production (Gadani et al 2012; Guo et al 2024)
Neuronal Regulation	Neuronal cells also play a role in regulating neuroinflammation. IL-4 released by the neurons, during any brain insult or in response to stimuli like LPS, can upregulate CD200 expression on neurons. This, in turn, attenuates the production of pro-inflammatory cytokines by glial cells. (Lyons et al 2009; Jonas et al. 1993)

Recovery from functional, cognitive and biochemical disorders due to neuroinflammation might become easier by understanding the role of cells of the immune system, mechanisms and duration of the process (Healy et al 2022). The four hallmark characteristics of neuroinflammation include increased proinflammatory cytokines, activation of glial cells, infiltration of peripheral immune cells and tissue damage (Mittli 2023; Estes and McAllister 2014). When the homeostasis of the brain is disturbed by various tissue insults, those factors which regulate inflammation get activated causing an acute reaction, in order to initiate the healing process. In the initial phase, this protective mechanism is facilitated by glial activation, which is stimulated by external as well as internal triggering factors (Leyane et al 2022; Lam et al 2021). On the other hand, if damaging factors strike repeatedly the acute inflammatory response of the brain becomes chronic, accelerating glial cell activation, eventually resulting in tissue fragmentation and degeneration. The positive feedback loop gets converted into a negative loop during chronic neuroinflammation, wherein the pro-inflammatory cytokines, ROS and RNS (reactive nitrogen species) are released in large number, and they further accelerate glial activation, leading to excessive level of inflammatory mediators (Lecca et al 2022). This escalates the rate of neuroinflammation by further increasing the infiltration of immune cells through the blood–brain-barrier. Prolonged activation of microglia, associated with abnormal protein aggregation, mitochondrial damage, axonal transport abnormalities, and neuronal apoptosis facilitate neurodegeneration, leading to impaired neuronal function. Microglia and astrocytes generate cytokines and chemokines, which are taken up by the active components of the neuroinflammatory process (Zang et al 2022). Mainly elderly people suffer from neurodegenerative changes because the major drawback of human aging is increased release of inflammatory cytokines, like IL-1 & 6 as well as TNF-α. These cytokines are directly linked to compromising ROS elimination mechanisms resulting in oxidative stress and stimulation of the major inflammatory response activator NFκB (Teleanu et al 2022). This explains the reason for persistent neuroinflammation which is the common feature in individuals with age-related neurodegenerative diseases.

## Blood–brain-barrier permeability facilitating neuroinflammation

Prolonged peripheral inflammation in the peripheral organs due to various tissue insults like colitis, pancreatitis, gastritis etc. can cause systemic inflammation with simultaneous enhancement of proinflammatory mediators in circulation, that in due course can lead to neuroinflammation (Sun et al 2022). Moreover, peripheral inflammation can also result from an imbalance in gut microbiota and other chronic infections (Mou et al 2022; Furman et al 2019). Peripheral inflammation leading to neuroinflammation is mainly due to the breakdown of the BBB through which peripheral immune cells infiltrate into the brain (Huang et al 2021). Disruption of the BBB takes place through different mechanisms involving the cytokines, certain proteins at tight junctions (TJ), endothelial changes as well as oxidative stress.

- At tight junctions between the endothelial cells the TJ proteins get degraded or disorganized by the excessive proinflammatory cytokines in the circulatory system, and this in turn disrupts the BBB. Though various inflammatory cytokines are responsible for TJ protein impairment downregulation of Claudin 5, a major TJ protein responsible for selective permeability of BBB, is the major causative factors for barrier disruption (Wang et al 2017; Nitta et al 2003).
- Peripheral inflammation also damages the endothelial cell of the BBB by inhibition of P-gp protein, a critical BBB transporter, activity that results in membrane abnormalities, damage the endoplasmic reticulum and mitochondria, ultimately leading to cell apoptosis (Cardoso et al 2012; Bernhart et al 2018). In Parkinson’s Disease vascular inflammatory markers were unusually higher in peripheral circulation (Yu et al 2020). This was reported to be due to the upregulation of Vascular Cell Adhesion Molecule 1 and Intercellular Adhesion Molecule 1, disturbing the barrier.
- Glial cell activation plays a substantial role in maintaining the integrity of BBB (Haruwaka et al 2019).

The consequence of peripheral inflammation on different neurological diseases varies, subject to the duration of inflammation and nature of disease.

- In multiple sclerosis (MS), the dysfunction of the BBB is triggered by oxidative stress and neuroinflammation causing TJ protein alterations and cytoskeletal changes. In addition, modulation of Mfsd2a gene, a major transmembrane protein present in the blood vessels, can upset the BBB (Ortiz et al 2014). This was witnessed when ablation of Mfsd2a gene in mice demonstrated a leaky BBB from embryonic stage to adulthood (Ben-Zvi et al 2014). Subsequent to a ruptured BBB, peripheral monocytes permeate into the site and begin to phagocytose myelin causing demyelination.
- Alzheimer’s disease is mainly associated with amyloid plaque deposition and microglial cells play a key role in amyloid protein processing (APP) and dysregulation of it causes plaque deposition. The resulting Aβ oligomers again activate the microglia and perivascular cells further to generate ROS. The resulting oxidative stress downregulates tight junction proteins and disrupts the BBB (Sweeney et al 2018). Microglial Toll Like Receptor-4 is instrumental in APP and the Lyn Kinase protein in it modulates plaque formation. Absence of Lyn protein in mice increased Aβ phagocytosis and decreased neuronal dystrophy, as observed by Islam et al (2025).

## Acute neuroinflammation

Acute neuroinflammation is an adaptive physiological immune response to protect and preserve the integrity of the central nervous system in response to injuries (Wyss-Coray and Mucke 2002). This healing process is mediated by a complex series of mechanisms at the cellular as well as the molecular level, primarily revolving around microglia and astrocytes (Ransohoff and Brown 2012). Key role of acute neuroinflammation is phagocytic clearance of apoptotic neurons, infection mediators and cell debris to prevent accumulation of neurotoxic elements that can further damage the surrounding nervous tissue (Wang et al 2022). Moreover, acute neuroinflammation often stimulates neurogenesis through growth factor (BDNF) upregulation, to help in the functional recovery of the affected region of the brain (Numakawa et al 2018).

## Chronic neuroinflamamation

When the neuroinflammatory process gets prolonged due to constant or recurring insults it becomes chronic, and it is instigated by a cascade of deleterious mechanisms mediated by the excessive activation of the same glial cells which were initially protective (Kempuraj et al 2017). Prolonged activation of microglia and astrocytes escalates the expression of proinflammatory cytokines, like TNF α, IL 1β, etc. (Simpson and Oliver 2020). This, in turn, amplifies oxidative stress and inflammation leading to chronic neuroinflammation and associated progression of neurodegenerative disorders (Teleanu et al 2022).

### Role of biomarkers and imaging techniques to differentiate acute and chronic neuroinflammation

Since the mechanisms of acute and chronic neuroinflammation vary in different neurological disorders, specific biomarkers might help to elucidate the pathophysiology of the diseases and subsequent development of novel therapeutic approaches. Existing clinical as well as pre-clinical approaches are not so efficient to differentiate adaptive immune activation (crucial for tissue repair and neuroprotection) from maladaptive inflammatory cascades that exacerbate neuronal injury and promote neurodegeneration (Doty et al 2015; Angiulli et al, 2021). Essentially, the development of specific biomarkers (molecular as well as neuroimaging) to analyze the inflammatory process under this context, would fortify the effectiveness of therapeutic strategies. Most frequently used biomarkers for acute neuroinflammation include C-Reactive protein (CRP) serum amyloid A and fibrinogen, procalcitonin and cytokines such as IL-6, IL1β and TNFα or transcription factor like NF-κB (Menzel et al 2021; Lelubre et al 2013). However, chronic neuroinflammation is usually associated with sustained activation of glial cells and subsequently elevated proinflammatory cytokines. Therefore, estimating Glial Fibrillary Acidic Protein (GFAP), Neurofilament Light Chain (NF-L) in axons, monocyte chemoattractant protein-1 (MCP-1/CCL2), transforming growth factor-beta (TGF-β) and YKL-40, the glycoprotein that is released by glial cells in their active state are helpful in differentiating chronic from acute neuroinflammation (Roveta et al 2024; Olsson et al 2016; Kwon et al, 2019). Recently, the biomarker GlycA, a proton signal measured through nuclear magnetic resonance (NMR), is emerging as a novel marker for neurological disorders (Huckvale et al 2020; Duprez et al, 2019). Since microglial activation is the hallmark in the etiology of neuroinflammation (Leng and Edison 2021; Heneka et al 2014), among the imaging techniques position emission tomography (PET) is a widely used method to visualize microglial activation. It utilizes translocator protein (TSPO), particularly 18kDa transmembrane protein as the probe (Zhang et al 2024). TSPO is positioned in the outer membrane of mitochondria of microglia, mainly involved in cholesterol transport and energy metabolism (Venneti et al 2006). The expression of TSPO is upregulated during neuroinflammation, which is relatively low under normal conditions and, therefore, it makes it a potential biomarker to investigate the progression of inflammation-linked neurological diseases. The progression of various neurological disorders that can be analyzed with PET are traumatic brain injury (Wang et al 2014), Alzheimer’s Disease (Liu et al 2015), Huntington’s disease (Simmons et al 2018), Parkinson’s disease (Rodríguez-Chinchilla et al 2020) and multiple sclerosis (de Paula Faria et al, 2014). Among the other emerging techniques, Magnetic Resonance Spectroscopy (MRS)and Diffusion Basis Spectrum. Imaging (DBSI) shows promising results in assessing cellular as well as metabolic alterations associated with neuroinflammation (Sun et al 2025). DBSI has been extensively validated in the brain to detect increased cellularity during inflammation demyelination and axon injury (Wang et al 2011, 2015).

## Role of different cells in neuroinflammation (Table 1)

Neuroinflammation is primarily driven by reactive astrocytes and microglia as well as peripheral proinflammatory cells that, after receiving signals, can cross the BBB and enter the CNS (Gullotta et al 2023). The major setback of chronic neuroinflammation is cognitive impairment that might affect the individual’s quality of life. It is a well-known fact that AD, one of the widespread neurodegenerative diseases, is characterized by learning impairment and memory incapability. This cognitive deficit associated with AD is believed to be due to chronic inflammation and AD exhibits a definite pattern of neuropathology like densely packed highly activated microglia and astrocytes along with elevated proinflammatory cytokines (McGeer and McGeer 1998; Akiyama et al 2000).

## Microglia

Microglia are derived from monocyte precursor cells and are known to be the brain-resident macrophages. Microglia mainly act like brain macrophages by removing cell debris, unnecessary synapses, microbes and other redundant substances and stabilize the microenvironment of the central nervous system (Ginhoux et al 2013; Ueno et al 2013; Araujo and Cotman 1992). Also, microglia contribute significantly during neurogenesis by maintaining neuron health and formation of the neuronal circuit. Under normal conditions, the microglia safeguard neuronal survival and provide immunity. They display dynamic adaptation in their shape from a ramified (resting phase) one to an amoeboid like (active phase), depending upon the normal physiological or pathological stimulation (Nimmerjahn et al 2005). Moreover, excessive aggregation of amyloid β leads to over-activation of microglia and consecutively undue release of pro-inflammatory cytokines and now they are referred as DAM—disease-associated microglia (Wolf et al 2017). Studies have proven that the binding of TLR4 and Aβ activates microglia, and the pattern recognition receptors help to detect the Aβ (Jin et al 2008; Tahara et al 2006). In mice model of AD, an increase in inflammatory cytokines production, phagocytosis inhibition and higher plaque accumulation was observed to be mediated through TLR4 signalling. Upon the inception of inflammation due to traumatic brain injury, microglia interact with the astrocyte to induce astrocyte-derived ATP gradient, which regulates the dynamics of microglial processes. Subsequently, these processes rapidly converge at the site of injury and help to establish a barrier between injured and healthy brain tissue (Davalos et al 2005; Haynes et al 2006). Another protective role of microglia is reported by Lou et al., wherein these glial cells facilitate repairing the breached BBB through purinergic receptor-mediated process (Lou et al 2016). However, when there is prolonged injury chronic inflammation sets in and the microglial processes retract due to the downregulation of purinergic receptor-mediated pathway and upregulation of the A (2A) adenosine receptor (Orr et al 2009). Additionally, this injury activated microglia secrete pro-inflammatory cytokines that can induce neurotoxic reactive A1 astrocytes (Liddelow et al 2017). These reactive A1 astrocytes outrun their normal beneficial function and become cytotoxic, which empowers them to destroy neurons & oligodendrocytes. Thus, the activated microglia boost neurodegeneration in several neurodegenerative disorders.

## Polarization of microglia

Triggered by brain injuries or infections microglia get activated and polarized into M1 or M2 types through different factors. M2 is neuroprotective, responsible for the release of anti-inflammatory cytokines, transforming growth factor (TGF β1) and promote the healing process (Gao et al 2023; Ronaldson and Davis 2020). Meanwhile, when the injurious state becomes severe microglial cells transform into M1 phase, which is proinflammatory, can lead to neuronal damage through the release of inflammatory cytokines, change tight junctions and modulate P-gp protein at the BBB. Their appearance changes to amoeboid shape with large cell body and a number of processes (Umpierre and Wu, 2021; Young and Morrison, 2018). Unwarranted over-activation of M1 microglia exert deleterious effects in neurodegenerative disorders (Tang and Le, 2016). In summary, M1 and M2 microglia switch between functional phenotypes according to different environmental stimuli.

## Astrocytes

The most abundant of glial cells that are derived from the neural stem cells are the astrocytes (Kriegstein et al. 2009). These cells are crucial in modulating extracellular balance of ions, fluids, removal of free radicals at the BBB as well as synapse formation and synaptic plasticity. In tripartite synapses, astrocytes are of paramount importance in bidirectional communication between them and neurons, which helps in preserving homeostasis and neuronal survival in addition to inter-neuronal communication (Farhy-Tselnicker and Allen 2018). Rodent study model of AD has exhibited reactive astrocytes around amyloid plaque (Kato et al 1998; Wegiel et al 2001). An elevated expression of glial fibrillary acidic protein (GFAP) and vimentin, both are proteins found in reactive astrocytes, were observed during neuronal cell hypertrophy. On the contrary, studies in the APP/PS1 mouse model of AD showed that the decrease of GFAP and vimentin is directly proportional to the astrocytic reaction and inversely proportional to that of the Aβ plaque load (Kraft et al 2013). It is demonstrated through in vitro and in vivo studies that reactive astrocytes form a glial scar and infiltrate Aβ plaque to decrease the damage of the neurotoxicity as well as reduce Aβ deposits by phagocytosis suggesting that it is neuroprotective (Wyss-Coray et al 2003; Xiao et al 2014). Gomez-Arboledas et al, (2018) claim that pathological changes such as impaired neural circuit, inflammatory responses and Aβ-mediated pathology can be reversed by improving the phagocytic potential of astrocytes. As mentioned earlier the injury activated microglia induce the generation of reactive A1 astrocytes which can further aggravate inflammation (Liddelow et al 2017).

### Polarization of astrocytes

****Like the microglia, astrocyte also aid in maintaining the homeostasis of the CNS. They maintain the blood–brain barrier (BBB), supply energy, recycle the metabolites, support the growth factors and regulate synaptic plasticity (Fan and Huo, 2021).

Astrocytes respond to various brain insults, undergoing morphological and functional changes, which is collectively termed as astrocyte reactivity and they are two polarities or phenotypes of astrocytes, such as A1 (neurotoxic) & A2 (neuroprotective) (Sofroniew 2020). The microglial cells get activated quickly when triggered by various brain insults, interact with the astrocyte to enable astrocyte reactivity and coordinate polarization of astrocytes (A1/A2) (Kwon and Koh, 2020; Liu et al 2020)). This is followed by the astrocytes modulating the microglial activation through complement proteins, cytokines, chemokines, etc. in a cyclical manner. This cross-talk between these glial cells exacerbates neuroinflammation.

Reactive astrocytes either aggravate neuroinflammation or provide beneficial anti-inflammatory effect, depending on their polarization as reported by Liddlelow et al., (2017). According to them, the A1 astrocytes are the transformed normal astrocytes that is triggered by the cytokines released from M1 microglia. These converted astrocytes lose their function like their ability to promote neuronal survival, formation of synapses and phagocytosis. They might also secrete a neurotoxin that destroys certain neurons and oligodendrocytes.

## Oligodendrocytes

Myelin loss and oligodendrocyte dysfunction are important variables that facilitate the progression of degenerative disorders of the brain (Han et al 2022). This malfunction sets off a series of events that eventually result in axon degeneration and neuronal cell death. These events include increased oxidative stress, mitochondrial failure, and ion channel redistribution along with denuded axons. Viral infections, acute demyelinating encephalomyelitis, and chronic illnesses like MS can cause impaired oligodendrocytes, which are evident in inflammatory disorders of the neurological system (Peferoen et al 2014). Beyond their role in myelination, oligodendrocytes are vital for ensuring axonal integrity, supporting axonal metabolism, and facilitating neuronal survival (Han et al 2022; Peferoen et al 2014; Li and Sheng 2023; Bankston et al 2013; Funfschilling et al 2012;). Oligodendrocytes can produce immune mediators in response to stress, have the ability to alter the microglial activation status, which in turn releases chemo attractants like CXCL10, CCL2, and CCL3 to recruit microglia to damaged tissues (Balabanov et al 2007; Ramesh et al 2012). Additionally, oligodendrocytes express beta-crystallin, a heat-shock protein, that activates microglia and plays a role in the pathophysiology of neurodegenerative illnesses like multiple sclerosis (Peferoen et al 2014; Balabanov et al 2007; Ramesh et al 2012).

## Peripheral immune cells

Antigen-presenting cells (APC), BBB permiability and lymphatics are primary defence mechanism that makes the brain an immune–privileged organ (Cheng et al 2023; Zhang et al 2019; Chen et al 2021; Parvez et al 2022). These systems collectively maintain CNS homeostasis and protect against peripheral immune system invasions. However, in pathological conditions such as stroke, these defences can be compromised. When the integrity of the BBB is disturbed, peripheral immune cells can infiltrate the affected brain regions. This invasion is exacerbated by the subsequent drainage of these cells through the CSF into the deep cervical lymph nodes, thereby amplifying the immune response (Cheng et al 2023; Cho et al 2022). Notably, peripheral immune cells have been observed within the brain parenchyma during the onset of stroke, highlighting the significant breach in the brain’s protective barriers. In essence, following a deleterious insult or pathology, the destruction of the BBB enables the entry of harmful substances and APCs into the CNS. This breach facilitates the recruitment of peripheral immune cells, which in turn propagates neuroinflammation (Cheng et al 2023). Consequently, the progression of neurodegenerative diseases is significantly influenced by peripheral immune cells.

## Insight into the mechanism of neuroinflammation

Multiple factors contribute to neuroinflammation that might result in neurodegeneration (Chang RC, 2009; Lyons A, 2009). Numerous epidemiologic, clinical, and animal model studies conducted over the past decades advocate that in most cases the CNS insult is associated with the glial cell activation, mainly astrocyte and microglia, along with proinflammatory cytokine rise. The neuroinflammatory process involves three major steps: the microglia and the resident macrophages facilitate inborn immune responses (Chausse et al 2021), the peripheral immune cells infiltrate into the CNS (Castellani et al 2023), and such infiltrated T-cells provide adaptive immunity (Korn and Kallie 2017; Gate et al 2020; Congdon et al 2019). Microglial cells are vital in the maintenance of brain homeostasis and support brain development (Chang et al 2009; Lyons et al 2009). However, the astrocytes are mainly responsible for the smooth circulation of blood, and they also supervise the optimum neurotransmitter level to provide an ideal microenvironment for neurological function. In the absence of inflammatory triggers, the anti-inflammatory cytokines, neurotrophic factors, or intercellular contacts supresses overactivation of these glial cells via CD200/300 receptor interactions, neurotransmitters, neurotrophic factors, and anti-inflammatory cytokines.

## Inflammatory cytokines in modulation of synaptic plasticity

Role of proinflammatory cytokines (TNF-α & IL-1β & 6) in modulation of synaptic plasticity is well known (Singh et al. 2024a; Heir and Stellwagen 2020). Among these cytokines, TNF-α exerts its effect on synaptic plasticity in a concentration dependent manner. Low or optimal level of TNF-α facilitates synaptic plasticity and substantially increased level of TNF-α as in conditions of neuroinflammation impairs the long-term potentiation (LTP) of excitatory neurotransmission leading to dysregulation of synaptic transmission and plasticity (Maggio and Vlachos 2018). Additionally, increased microglial activation during neuroinflammation adds to the pathological enhancement in TNF-α concentration, since microglia is known as the major source of this cytokine (Shemer et al 2020). TNFα acts on astrocytes to modulate synaptic neurotransmission, providing evidence for a relevant crosstalk between glial cells (Santello et al 2011). Moreover, TNF-α affects neuroplasticity by altering the glutamatergic pathway, wherein it alters the expression of glutamate receptors. It intensifies the internalization of AMPA receptors and interrupts the functioning of NMDA receptors. This causes diminished LTP, which is essential for cellular correlation of learning and memory (Henley et al. 2013). Among other cytokines, the IL-1β hampers LTP by altering BDNF-TrkB (Tyrosine kinase B) signaling that is essential for strengthening the synapses. It also triggers stress related p38 MAPK (Mitogen activated protein kinase) that negatively affects neurogenesis in the dentate gyrus (Tong et al. 2012). Elevated expression of IL-6 during neuroinflammation adversely affects JAK/STAT3 signaling pathway, the vital pathway for regulating neuronal growth and survival (Lee et al 2016). It also decreases the synaptic protein expression, disrupting glutamatergic transmission and thereby dysregulating synaptic plasticity (Gruol 2015).

## Genetic factors modulating neuroinflammation

Identifying the molecular mechanisms of the specific gene might help in minimizing the progression of neurodegenerative disorders significantly. Though there are multiple factors involved in neurodegenerative diseases, the contribution of genetic factors cannot be ignored since Apolipoprotein E (APOE) gene variant-related pathway amplified the risk of late-onset AD (Heneka 2023; Rosenthal and Kamboh, 2014, Yamazaki et al 2016). APOE variants increase the risk of Aβ protein aggregation and tau pathology in both human (Hashimoto et al 2012; Koffie et al 2012) animal model (Youmans et al 2012). Among the variants, the APOE4 & TREM2 can exacerbate neuroinflammation by escalating the release of pro-inflammatory cytokines by activated glial cells. It also impedes amyloid β clearance, which can further accelerate microglial activation, eventually leading to impaired synaptic plasticity and cognitive deficit (Heneka et al., 2023).

These variants are associated with a distinct microglial activation state termed “disease-associated microglia” (DAM), which initially serves a protective role but may become maladaptive with prolonged activation (Keren-Shaul et al. 2017).

## Neuroinflammation on neurodegeneration and associated cognitive impairment

The pathological sequence of neurodegeneration is a cycle where mainly amyloid and tau protein aggregates induce neuroinflammation, followed by neurodegeneration and cognitive impairment, which further facilitates protein aggregation worsening the condition. In the aged population other than the increased release of neuroinflammatory cytokines due to various influences, the hormonal imbalance also plays a pivotal role. In women decline in estrogen level increased the risk of AD and associated cognitive impairment, witnessed in both human and animal studies (Li et al 2014; Vegeto et al 2006; Yue et al 2005). Whereas males have a higher risk of Parkinson’s disease, in comparison to females (Wooten et al 2004).

## Age and sex differences

Older adults are usually more prone to neurodegenerative diseases (NDs), since advancing age is the major risk factor for inflammation-induced neurodegeneration. This can be credited to increased pro-inflammatory cytokines, tau and amyloid aggregation, impaired mitochondrial function in response to various types of tissue insults during their life-time (Azam et al. 2021). Similarly, progression of several NDs such as Alzheimer’s, Parkinson’s, MCI, depression or Amyotrophic Lateral Sclerosis exhibits gender differences. Thes can be attributed to hormonal influence or genetic factors like APOE protein variant (Nicoletti et al 2023). Genetic variation in immune-associated signals may contribute to altering the expression and function of immune signals independent of pathogen exposure (Sekar et al 2016).

## Sex hormones on neurodegeneration

The sex hormones play a significant role in neurodegenerative diseases like AD, PD etc. (Ardekani et al. 2016). *In-Vitro* studies have revealed that sex hormones, testosterone and estradiol exert varied effect on the cleavage of tau proteins (Yeap and Flicker 2022). Since estrogen has a neuroprotective property in women, a decline in estrogen level is associated with AD (Villa et al 2016; Wise et al 2001). Therefore, the risk of AD development in perimenopausal women is more than the elderly men (Green and Simpkins 2000). In a transgenic mouse model overexpressing amyloid precursor protein, downregulating aromatase expression increased testosterone and reduced estradiol concentrations, and reduced plaque formation within the brain, inferring a role for testosterone rather than estradiol to protect against Alzheimer’s disease (Yeap and Flicker 2022; McAllister et al 2010). In vivo studies provided further, strong evidence on the capacity of estrogens to inhibit neuroinflammatory processes, by modulating the receptors in microglia (Lei et al 2003).

Whereas males have a higher risk of Parkinson’s disease (PD), in comparison to females (Wooten et al 2004). In PD, the neurons containing α-Syn aggregates in Lewy bodies cause inflammation and neuron degeneration and produce motor as well as cognitive disorders (Yeap and Flicker 2022). Therefore, inhibition of α-Syn aggregation is considered a promising therapeutic strategy for PD and several other synucleinopathies disorders that are leading causes of dementia worldwide (Khan et al 2024).

Testosterone upregulates glial cell line-derived neurotrophic factor (GDNF) in glioma cells and astrocytes essential for microglial proliferation, migration, and invasion. It also contributes to brain tumor growth via GDNF and inflammation. Testosterone is a crucial hormone that regulates the central nervous system diseases such as brain and spinal cancers by activating oncogenes, pro-inflammatory cytokines, and chemokine synthesis that facilitates astrocyte reactivity, cancer cell proliferation, and tumor progression (Kanwore et al 2023).

Recent studies have shown the link between androgen deprivation therapy (ADT) for prostate cancer and Alzheimer’s disease in older people (Sari Motlagh et al 2021; Hwang et al 2015; Nead et al 2016). Modulation of testosterone, as in prostate cancer treatment, can lead to amyloid aggregation and formation of neurofibrillary tangles, by activating neprilysin (NEP) and eventually cause cognitive decline. Neprilysin is the protein responsible for regulating the accumulation of amyloid β oligomers into amyloid plaques (Qian et al 2021). Alterations in basal testosterone expression can disturb the stability of genes that are sensitive to androgen level, contributing to cognitive deficit (Singh et al. 2024b).

## Impact of neuroinflammation on neurotransmitters

The proinflammatory cytokines released by the activated microglial cells in the course of neuroinflammation are capable of altering neurotransmitter production and receptor sensitivity (Salcudean et al. 2025; Wang et al 2022).

## Serotonin

Several studies have shown the connection between neuroinflammation and neuropsychiatric disorders like Major Depressive Disorder (MDD) & Parkinson’s disease which is associated with cognitive disturbance (Robson et al 2017; Remus and Dantzer, 2016). Neurotransmitter serotonin is often associated with mood regulation and is associated with depressive disorder. However, there is no direct link between serotonin depletion and depression (Moncrieff et al 2023). On the other hand, tryptophan (the precursor of serotonin) metabolite kynurenine produced by the enzyme indoleamine 2,3 dioxygenase (IDO) is secondarily involved in cognitive disorder (Wu et al 2019). The Kynurenine mediated pathway other than disturbing serotonergic systems, also leads to deposition of neurotoxic metabolitess such as quinolinic acid, which is known to augment NMDA receptor mediated excitotoxicity (Muneer 2020). IDO is secreted by the microglia, induced by the cytokine interferon-γ. Interestingly, IDO expression was observed to be higher in the hippocampus of mice model of AD and AD patients, along with neurofibrillary tangle and amyloid plaques (Wu et al 2013; Willette et al 2021). Reduction of tryptophan by increased expression of IDO during neuroinflammation might lead to serotonin depletion leading to mood dysregulation (Remus and Dantzer 2016).

## Dopamine

Another neurotransmitter dopamine regulates neuroinflammation, influencing microglial activation and supressing activation of NLRP3 inflammasome (Iliopoulou et al 2021; Yan et al 2015), through various dopamine receptors (DR1-DR5) expressed in the immune system. Among these receptors, DR1 and 2 take part in anti-inflammatory mechanism, reducing neuroinflammation (Montoya et al 2019; Wang et al 2018).Whereas DR3 and DR5-associated signaling pathway facilitate neuroinflammation (Contreras et al 2016; Osorio-Barrios et al 2018). In this regard, it has been shown that human microglia express DRD1-DRD4 (Masteroeni et al. 2009), whilst DRD1, DRD2, DRD4 and DRD5 have been found in rat microglia (Farber et al 2005).

## Glutamate

The influence of neurotransmitter glutamate associated pathway is crucial for maintaining the integrity of neuronal cell membrane and there by facilitating appropriate neurological function.

(Sears and Hewett 2021). Inhibitory signals in the path neuronal transmission are mediated by GABA, while excitatory signals are mediated by glutamate, any imbalance in this circuit is known to cause excitotoxic injury in hippocampus, the memory-associated region (Zhou et al. 2021; Takeuchi 2010). Activated microglia during chronic neuroinflammation modulates glutamate synthesis and disruption of the balance between glutamate and GABA has a functional role in various pathologies, including traumatic brain injury, Alzheimer’s disease, epilepsy, schizophrenia, and depression (Sears and Hewett 2021; Wong et al 2003).

## Neuroinflammation in major areas of brain associated with cognition

Among the different regions of brain hippocampus, prefrontal cortex and amygdala are relatively more vulnerable to neuroinflammatory damage, which can be attributed to their unique neuronal structure, metabolic needs and associated cognitive functions (Mittli et al. 2023). Recently, studies on microbial influence on neuroinflammation have shown that the antibiotic exposure as well as microbiome-derived interventions can exert region-specific effects on the brain, that might remain throughout one’s lifetime, to a certain extent (Sharvin et al 2023). Both human and animal studies, as shown in Tables 2, 3 & 4, have demonstrated solid involvement of the medial prefrontal cortex (mPFC), ventral hippocampus, and amygdala in the development of anxiety, depression, and associated behavioural regulation (Russo and Nestler 2013; Price and Drevets 2010). Enhanced proliferation and activation of microglia in above mentioned cognitive areas of the animal brain were observed when restraint stress, inevitable foot-shock stress and chronic unpredictable stress was applied to them in different studies (Frank et al 2007; Hinwood et al 2012; Kreisel et al 2014). However, in patients suffering from severe depression, microglia activation was observed in the insular cortex, anterior cingulate cortex, and cerebral cortex too (Steiner et al 2011; Schnieder et al 2014).

**Table 3:** studies on neuroinflammation in hippocampus

References	Method	Sample	Biochemical studies	Cognition assessment	Histological observation	
Izquierdo-Altarejos et al 2023	Hyperammonemic rats	Hippocampus	↑IL-1β ↑TNF-α	Memory deficit by Y-maze, Object recognition & Object location test	Micoglial & astrocyte activation	
Komoltsev et al 2021	Rat- lateral fluid percussion cortical injury	Hippocampus	↑IL-1β ↑corticosterone	Seizures	Neuronal loss, Microglial activation	
Blossom et al 2020	Restraint stress induced rats	Brain	↑serum cortisol ↑C-reactive protein (CRP)	–	Neuronal loss in prefrontal cortex & hippocampus	
Komoltsev et al 2020	Patients- acute traumatic brain injury	Electrocorticograms (ECoGs) & EEG	–	Epileptiform activity through ECoGs & “brush-like” ECoG pattern superimposed over rhythmic delta activity	–	
	Rat model of Alzheimer’s disease	hippocampus	↑IL-1β ↑IL-6 ↑TNF-α	Memory deficit by radial maze & elevated plus maze test	Neuronal loss in hippocampus	Neurodegeneration noted in hippocampus
Zhao et al 2020	Rat exposed to aluminium chloride	hippocampus	↑IL-1β ↑IL-6 ↑TNF-α	Memory impairment by water maze, elevated plus maze & open field test	Neuronal loss and vacuolar spacing in hippocampus	Neuron shrinkages and vacuole spacing around the neurons in hippocampusnoted
Chiroma et al 2019	Rat – aluminium Chloride exposure	Hippocampus	–	Cognitive impairment seen by T-maze, elevated plus maze and novel object recognition test	Neuronal loss in CA1	
Kumar et al 2019	Rat—aluminium Chloride exposure	Hippocampus	–	Memory impairment by passive avoidance test	Neuronal loss in CA1 & CA3	
Song et al 2018	Rat—LPS & chronic unpredicted mild stress	Brain	↑cyclooxygenase-2 ↑microglial & astrocyte activation	Forced swim test showed behavioural deficit	–	
Tyrtyshnaia et al 2016	LPS (*In-vitro & in-vivo*)	CA1 of hippocampus	↓intracellular pH leading to intracellular acidification of CA1 neurons	–	–	
Mandal et al 2012	Patients—novel multi-voxel 31P MRS imaging experimental scheme - advanced 31P signal processing technique	Hippocampus	↓phosphomonoesterase ↑phosphodiesterase in AD patients	Mild cognitive impairment	–	
Ziehn et al 2010	Experimental autoimmune encephalitis mice model	Hippocampus	–	Barnes Maze test revealed hippocampal-dependent learning & memory deficit	↓pyramidal cell layer volume in CA1 ↓GABA nergic inhibitory interneurons ↑neuronal and glial cell death

**Table 4:** studies on neuroinflammation in prefrontal cortex

References	Method	Biochemical studies	Cognition assessment	Histological observation
Massand et al 2024	Rats exposed to aluminium Chloride	↑IL-6 ↑MDA	–	Neuronal loss in prefrontal cortex
Yegla & Foster 2019	Rats exposed to LPS	↑IL-1α ↑IL-6	Cognitive slowing without motor impairment	Food restriction increased number of activated microglia in young rats whereas in middle-aged rats the number of microglia was reduced
Li et al 2022	Bone Cancer pain induced rats	↑expression of CD74 and CTSS in PFC,	↑Pain related anxiety along with walking disability in elevated plua maze experiment	↑microglial activation in PFC
de Pablos et al 2006	LPS and stress induced Rats	↑TNF α ↑IL6 ↑IL 1β	–	GABAergic Neuronal loss in PFC

## Hippocampus and neuroinflammation

### Anatomy of hippocampus

**Fig. 1:**
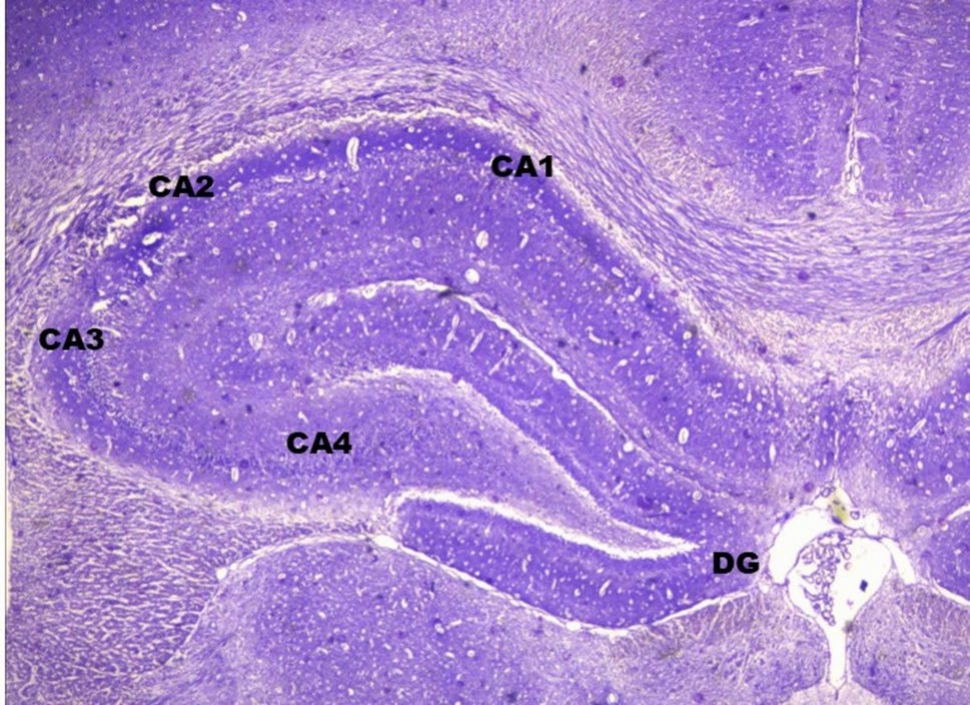
photomicrograph of Cresyl violet-stained dentate gyrus (DG) and cornu ammonis (CA) areas of hippocampus of left side rat brain under 20x

Hippocampus is a part of the limbic system, hidden on the medial aspect of the temporal lobe of the cerebrum (Tatu et al. 2014). The name is derived from its shape because of its resemblance to seahorse. Its different regions resemble ram’s horn (Ammon’s horn) in the coronal section of the brain, hence designated as cornu ammonis (CA) regions, because Amon is an Egyptian deity with ram’s head mythologically (Knierim 2015). Anatomically hippocampus is made up of the dentate gyrus, cornu ammonis (CA) and subiculum (Rao et al 2022; Parmar et al 2018). Cornu ammonis consists of four regions (CA1–CA4) and CA4 is continuous with the dentate gyrus (Fig. 1). It is a well-known fact that the hippocampus is implicated in cognitive functions & memory processing (Tatu et al. 2014). It mainly contributes to episodic memory as well as the regulation of emotion (Bartsch and Wulff 2015). The cognitive function of the hippocampus is performed through the Papez circuit wherein hippocampus is connected with the mamillary body and then through the mammillothalamic tract the information is projected to the anterior thalamic nuclei. Finally, the fibres relay in the cingulate gyrus completing the circuit (Papez 1937; Aggleton and Brown 1999). Before Papez credited the hippocampus and mamillary body with the role of emotion, both these structures were implicated in memory dysfunction. Hippocampal lesion was revealed in a patient who experienced amnesia after a stroke (Bechterew 1900) and earlier to it Brown and Schafer (1888) reported about impaired memory following hippocampal lesion in a rhesus monkey. Likewise, Gudden (1896) and Gamper (1928) identified atrophy of the mamillary body in Korsakoff’s syndrome, in which memory loss is the main feature.

### Neuroinflammation in hippocampus

**Fig. 2:**
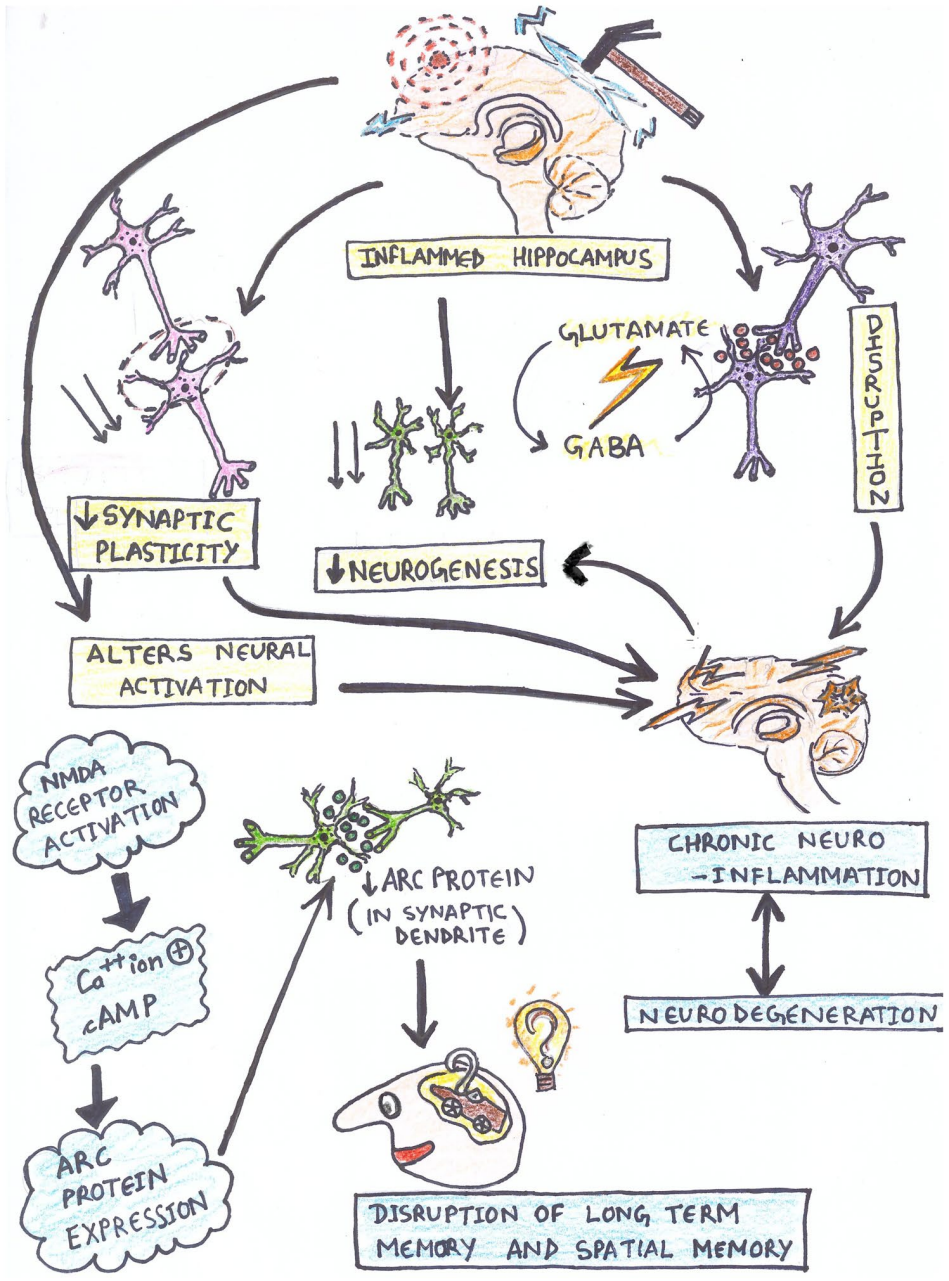
schematic representation of impact of neuroinflammation in hippocampus

Prolonged inflammation in hippocampus can affect synaptic plasticity, neurogenesis, and the stability of GABA & glutamate-like neurotransmitters (Guzowski et al 2000). Activation of hippocampal and parietal cortex neurons after a behavioural event is essential for long-term potentiation and spatial memory consolidation, which depend on Arc protein expression (Guzowski et al 2000). Thus, local neuroinflammation can alter neural network activation, potentially leading to learning and memory deficits associated with various neurological conditions (Fig. 2). Both preclinical and pathological data provide credibility to the idea that neuroinflammation modulates hippocampus function (Colasanti et al 2016). There are several studies revealing the effect of neuroinflammation in hippocampus affecting cognitive functions (Table 3).

High concentration of IL-1 receptors in the hippocampus makes it susceptible to neuroinflammatory stimuli (Green and Nolan 2014). Hippocampus is known for its ability to adapt to functional and structural changes (plasticity), and neuroinflammation negatively affects its plasticity (Weerasinghe Mudiyanselage et al. 2022; Cooper et al 2018). This is because neuroplasticity can be modulated by various factors like neurotrophins, neurotransmitters as well as cytokines and chemokines which can all be negatively influenced by neuroinflammation.

According to some studies development of depression is associated with prolonged neuroinflammation in cognitive areas of the brain (Wager-Smith and Markou 2011; Miller et al 2009). Pathological change in the hippocampus is linked to lipopolysaccharide-induced depressive like behaviour (Frenois et al 2007). In the rat model of experimental allergic encephalomyelitis (EAE), defective neurogenesis is linked to hippocampal neuroinflammation (Giannakopoulou et al 2013). A study on SARS-CoV-2 infected hamsters and COVID affected deceased patients revealed neuroinflammation that was evident in the form of an increased number of activated microglia and elevated interleukins like IL-1β & IL-6 in the hippocampus (Soung et al 2022). Repeated social defeat (RSD) in mice model induced neuroinflammation and caused spatial memory impairment. This was observed to be through enhanced activation of microglia in hippocampus, which escalated the release of proinflammatory cytokines and caused memory impairment (Garrido-Mesa et al 2013; McKim et al 2016; Pfau and Russo 2016).

Moreover, peripheral inflammation induced neuroinflammation can alter the neuronal architecture, expression of genes and neurotransmitter mediated pathway, especially serotogenic pathway in the hippocampus and amygdala under the influence of signals from gut microbes (Sharvin et al 2023). This crosstalk between peripheral and neuroinflammation is chiefly through the gut-brain-axis and is primarily by microglial activation (Erny et al 2015; Silva et al 2020; Mossad et al. 2020). In study models of AD (animals & humans), the changes in hippocampal architecture were accompanied by significantly altered composition of gut microbiota (Tang et al. 2020, Verhaar et al. 2022). This was witnessed when healthy mice exhibited microglia activation in the hippocampus along with cognitive deficit, when gut microbes of AD patients were transplanted to them.

## Prefrontal cortex (PFC) and neuroinflammation

### Anatomy and physiology of prefrontal cortex

**Fig. 3:**
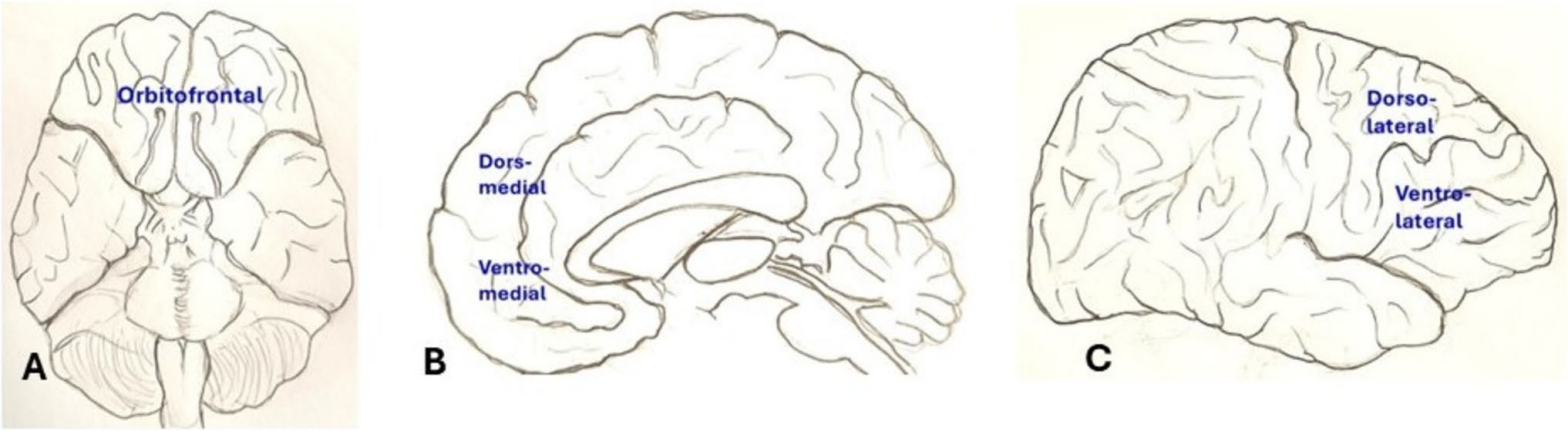
diagrammatic representation of different parts of prefrontal cortex (reproduced from Szczepanski and Knight 2014)

Prefrontal cortex is in the frontal lobe anterior to the premotor areas (Johns P 2014; Szczepanski and Knight 2014). It is further divided into dorsal (dorsolateral & dorsomedial), orbital and medial parts (Fig. 3). The prefrontal cortex functions as the executive hub of the brain, regulating working memory, attention, decision-making, behavioural inhibition, and cognitive flexibility (Teffer and Semendeferi 2012). The dorsolateral part is implicated in coordinating behavioural pattern and cognitive processes such as working memory, decision making and value encoding, wherein it prioritizes the received information depending upon its importance, relevant to the person’s need or goal (Szczepanski and Knight 2014; Seminowicz and Moayedi 2017). The orbital part deals with inhibitory action like checking and curbing the inappropriate behavior, especially during social interactions of the individual (Szczepanski and Knight 2014). The medial part is involved in controlling mood and motivation. It is also said to be a part of ‘default network’ of the brain that is activated during quiet observation and thought process. It is also reported that the medial and rostral prefrontal cortex is responsible for threat response and arousal and the lateral and caudal prefrontal cortex is responsible for reward learning and behavioural control (Huey 2015). Magnetic Resonance Imaging studies have revealed that PFC activity is vital for tasks requiring planning and outcome assessment (Friedman and Robbins 2022). Impaired functioning of PFC due to lack of coordination between neurotransmitters and neuronal circuits has been implicated in conditions like schizophrenia, depression and ADHD (Arnsten 2009). Enhanced cognitive performance by the prefrontal cortex was observed in clinical studies that involved neuromodulation (modulating brain activity) and cognitive training (Keshavan et al 2014; Antonenko et al. 2024; Matsuzaki et al 2023).

### Neuroinflammation in prefrontal cortex

**Fig. 4:**
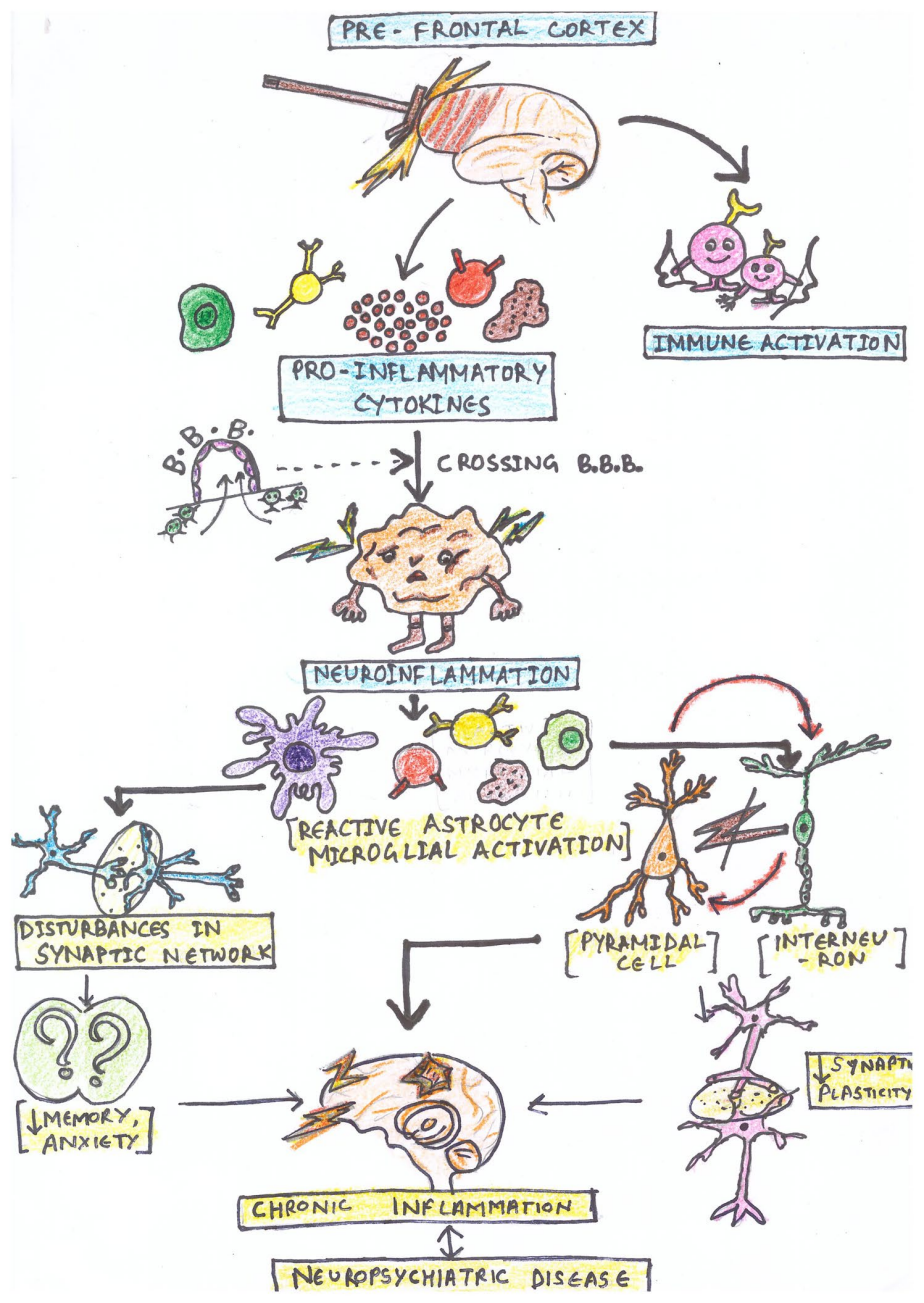
schematic representation of impact of neuroinflammation in prefrontal cortex

Studies have shown that the cognitive impairment observed in neurodegenerative disorders like AD, PD or infection-mediated developmental diseases of nervous system are linked to defective functioning of the medial prefrontal cortex (Arnsten et al. 2021; Ji et al 2020; Stanojlovic et al 2019; Richetto et al 2017). Peripheral immune challenges can cause complex proinflammatory changes, influencing the neuronal functions leading to neuropsychiatric symptoms like depression (Mittli 2023), schizophrenia, bipolar disorder (Huey 2015). Since it plays a prime role in learning and memory through its extensive connection, the development of mental comorbidities associated with chronic pain are also attributed to altered functioning of medial PFC (Condes-Lara et al., 1989; Hardy 1985). The functioning of PFC involves integrated activity of excitatory and inhibitory neurons (Mittli 2023) and inflammation brings about a change in this balanced activity. The imbalance in excitatory/inhibitory activity of neurons is the characteristic of several neuropsychiatric conditions, as schematically depicted in Fig. 4. On the contrary, acute neuroinflammation in PFC was beneficial in the novel object recognition memory test as it enhanced the recruitment of parvalbumin expressing (PV) interneurons in medial PFC (Feng et al 2021). Brain imaging data have revealed dynamic changes in the activity of the PFC in human subjects trying to manage stress (Sinha et al 2016). The cytokines in the PFC play a major role in neuronal activity & plasticity (Gamo and Arnsten 2011). Stress-induced neuroinflammation in PFC modulates glutamatergic system and, in turn, affects cognition (Jin et al 2018). Additionally, PFC can be affected by peripheral inflammation from dysbiosis in gut microbiota through gut-brain-axis. These microbes can influence myelination of axons (Hoban et al 2016), lipid metabolism (Chen et al 2019) as well as gene expression (Muhammad et al 2022; Sittipo et al 2022) within the PFC. A number of studies have shown peripheral inflammation leading to neuroinflammation in the prefrontal cortex, which was evident in the form of neuronal loss in the prefrontal cortex along with an increased number of activated microglia with a simultaneous increase in the expression of neuroinflammatory cytokines in the brain (Table 4).

## Amygdala and neuroinflammation

### Anatomy and physiology of amygdala

**Fig. 5:**
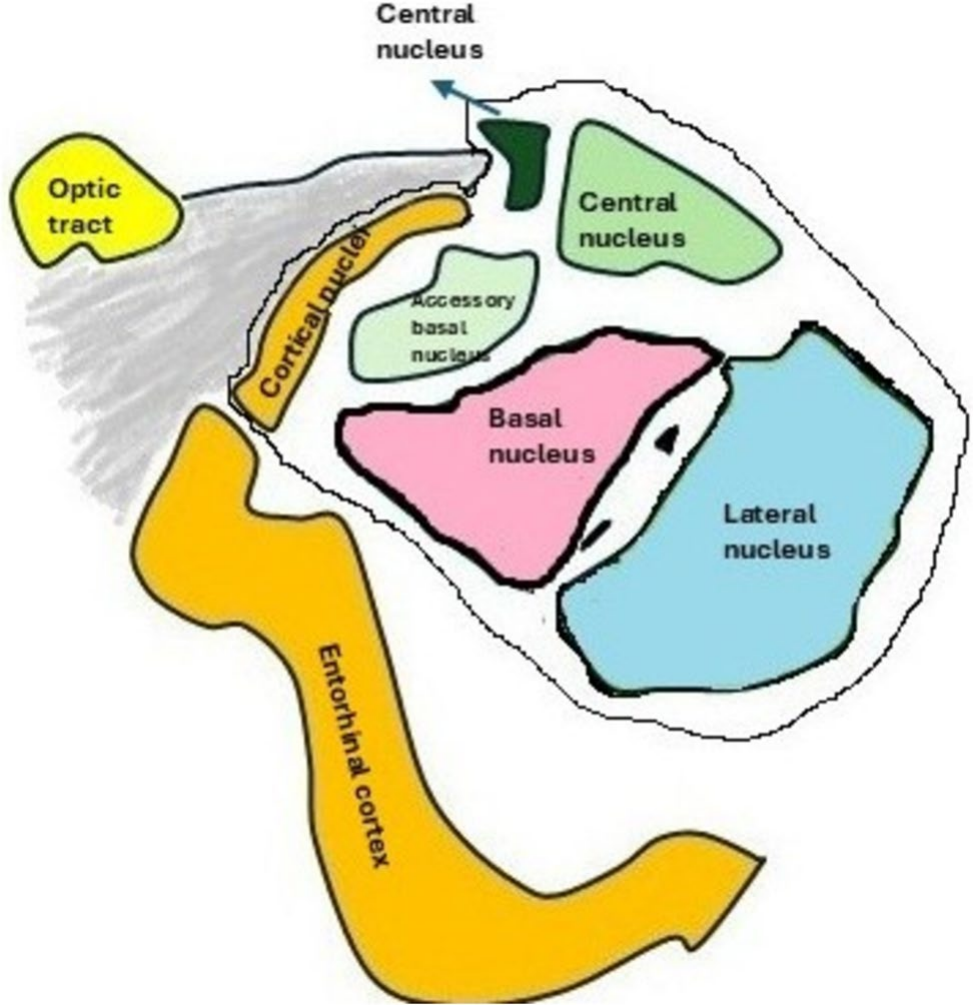
diagrammatic representation of different nuclei of amygdala (reproduced from Simic et al 2021)

Amygdala is a part of the limbic system, in the temporal lobe of the cerebrum. The name amygdala is derived from “almonds”, because of its shape (Rajmohan and Mohandas 2007). It is made up of around thirteen nuclei with a broad range of inter as well as intranuclear connections. The classification was initially introduced by Price et al. (1987) and later modified as (i) the deep or basolateral that includes lateral, basal and accessory basal nucleus (ii) the superficial or cortical-like group that includes cortical nuclei & nucleus of lateral olfactory tract and (iii) the centromedial group that comprises medial and central nucleus as depicted in Fig. 5 (Sah et al 2003). In addition to these three groups, another group of nuclei include the extended amygdala and amygdohippocampal area. Later Swanson and Petrovich suggested that the amygdalar complex should be divided into four functional systems, such as the frontotemporal, autonomic, main olfactory and accessory olfactory systems (Swanson 2000). The basolateral nuclei constitute the frontotemporal group, the centromedial nuclei form the autonomic group and the cortical-like nuclei constitute both olfactory & accessory groups, as proposed by Sah et al (2003). Amygdala plays a significant role in the fear-related response circuitry and in human, disturbances in this circuitry are likely to be the triggering factor in certain anxiety disorders like post-traumatic stress. Specific groups of nuclei of the amygdala have been assigned with particular function, mainly associated with stress. Accordingly, the basolateral nucleus manages behavioural & physiological responses to stress (Bhatnagar et al 2004). The central group responds to fear and drug-linked stimulants (Gilpin et al 2015). The extended amygdala known as the bed-nucleus of the stria terminalis is linked to anxiety (Li et al 2012). Along with processing fear and other aversive stimulations, amygdala is also associated with hunger stimulants (Sah et al 2003). In terms of memory function, the activated amygdala modulates the acquisition and coalition of memories, together evoking an emotional reaction. Through its connection with hippocampus, it acts like a store house of emotional memory like remembering birthday, anniversary etc. as well as fear memory like remembering the trauma experienced (Tang et al 2020). The amygdala is also connected with thalamus, hypothalamus, orbitofrontal as well as the anterior temporal cortex. The circuit that connects the amygdala with the thalamus and orbitofrontal cortex has been suggested as a pathway through which an individual can deduce people’s intentions through their body language and which helps in social interactions (Deakin et al 1989). Because of this extensive network amygdala has been linked to depression, sleep debt, anger and certain similar cognitive disorders (Ruiz et al 2018; Saghir et al 2018).

### Neuroinflammation in amygdala

**Fig. 6:**
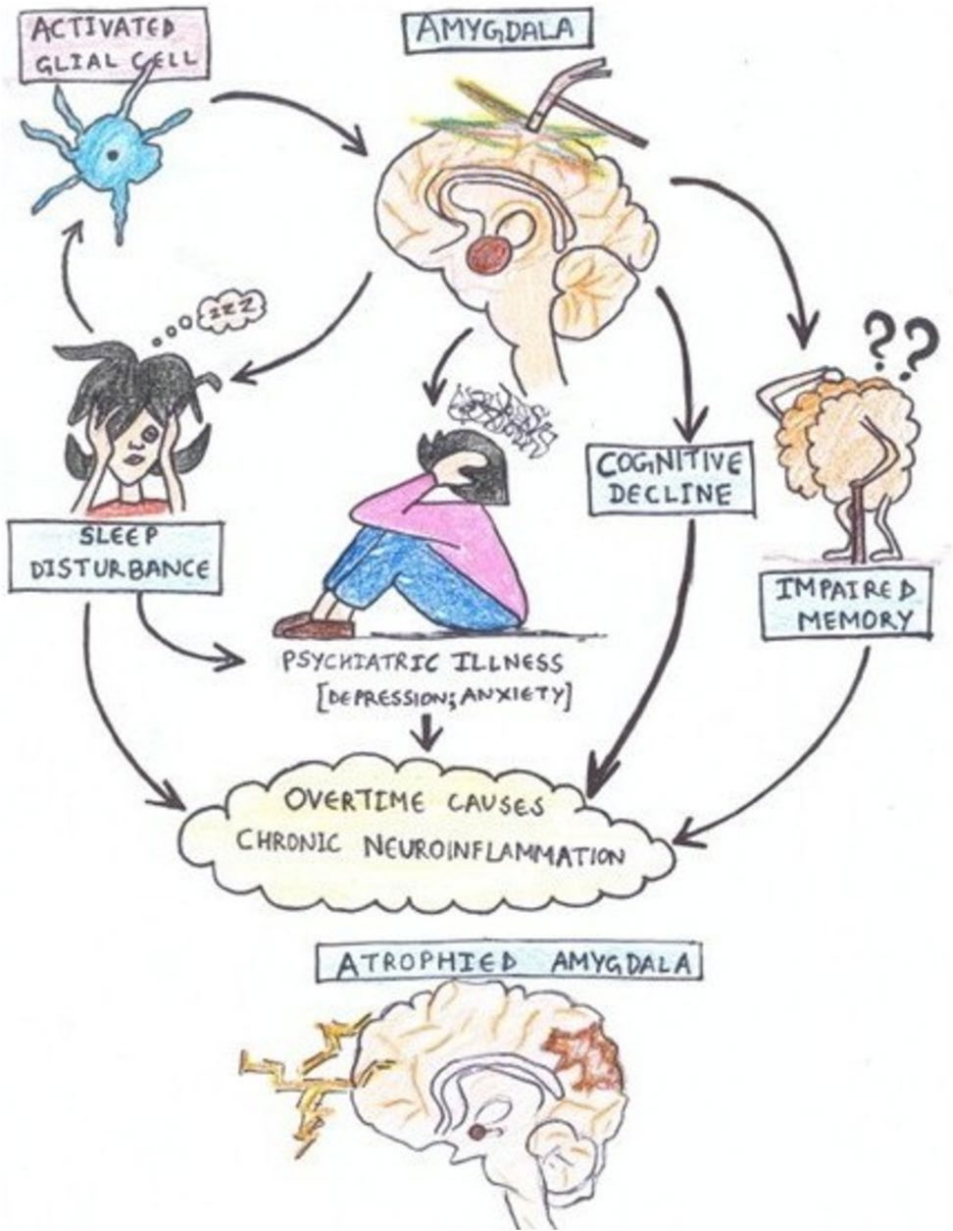
schematic representation of impact of neuroinflammation in amygdala

It is a well-known fact that chronic stress induces neuroinflammation (Hassamal 2023) and amygdala is the main region of the brain that mediates stress response and over stimulation of it results in anxiety and depression (Rosenkranz et al 2020; van den Bulk et al 2014). Few studies have shown the implication of neuroinflammation in amydala leading to cognitive decline and reduced amygdala volume leading to anxiety (Table 5). It is reported that at rest amygdala activity is significantly inhibited, while exposure to chronic stress causes over-activation of it, resulting in cognitive changes and this is mediated by suppression of GABA receptor currents (Xia et al. 2022). Such hyperactivation of amygdala due to stress might lead to psychiatric disorders and it was discovered that adolescents with depression had hyperactive amygdala (Yang et al 2010). Guo and co-authors (2023) have reported that social pressure-induced anxiety can induce neuroinflammation in individuals and this, in turn, can change the neural circuits related to amygdala, thereby further worsening neuroinflammation (Fig. 6). Threat-fear circuit, the neuronal circuit involved in anxiety disorders (Robinson et al 2019) mainly include prefrontal cortex, amygdala, hippocampus and other neighbouring regions of the brain (Xu et al 2019). Stimulation of the central amygdala and anterior cingulate gyrus led to a proinflammatory effect on gastric ulcers and inhibiting these regions prevented worsening of stress-related gastric inflammation, as reported by Henke et al (1982). They suggest that proinflammatory effect of the amygdala can also contribute to peripheral inflammation other than the brain. Generally, through the thalamus and cortical areas the external stimuli are conducted to amygdala (Guo et al 2023). This subcortical group of nuclei in the anterior part of the medial temporal lobe is involved in memory processing linked to emotion (Domínguez-Borràs and Vuilleumier 2022). There are studies proving that it is the core structure mediating stress response through its overactivation (Rosenkranz et al 2010; van den Bulk et al 2014). It is reported that inflammation in amygdala due to stress, plays significant role in the development of anxiety and depression (Tottenham and Sheridan 2010; Hu et al 2022). Neuronal functional alterations resulting from this inflammation may impact molecular signaling, cellular activity, and synaptic plasticity. Furthermore, the amygdala may undergo structural alterations as a result of prolonged stress and inflammation. According to Tottenham, stress can enlarge and hyperactivate the amygdala initially. But, over time, protracted stress and inflammation may cause cellular atrophy and decreased amygdala volume (Fig. 6). This pattern is frequently seen in people with persistent depression, who first exhibit an enlarged amygdala before developing a reduced volume because of ongoing stress and inflammation (Tottenham 2010). Long-term physical activity has been demonstrated to lessen the impact of stress and inflammation on the amygdala (Pahk 2023). Frequent exercise may have a protective effect against stress-related neurobiological alterations by lowering the metabolic activity of the amygdala and dissociating it from systemic inflammation. The anxiety-related behavioural changes like startling or freezing are associated with amygdala connections (Zhang et al 2023b). Amygdala, the centre of fear, is believed to trigger the autonomic nervous system during anxiety and the information is further relayed to the hippocampus and the basal nucleus. Particularly, lack of sleep significantly alters mood, memory, learning, cognition and decision-making capacity amongst other aspects of mental health. Sleep deprivation causes a functional deficit between the amygdala and the ventral anterior cingulate cortex (vACC), which can increase the amygdalar response to negative stimuli and a drop in mood (Motomura et al 2013). Neuroinflammation is one of the most frequent side effects of sleep deprivation (SD), which sets off a variety of inflammatory reactions (Mahalakshmi et al 2022). Higher and lower levels of inflammation are linked to SD-induced changes in the level of pro- and anti-inflammatory cytokines in the blood. The primary causes of neuroinflammation are BBB disruption, inflammatory cytokine release, poor glymphatic clearance, microgliosis, and astrogliosis brought on by SD. Emotional instability results from sleep debt because it impairs the medial prefrontal cortex's (MPFC) capacity to inhibit amygdala activity. It is demonstrated from a variety of animal models that stress causes the amygdala to become overactive (Rosenkranz et al 2010; Zhang and Rosenkranz 2012). Patients with stress or mood disorders also have increased amygdala activity, according to human neuroimaging evidence (Birbaumer et al 1998; Phan et al 2006). It is widely known that sleep loss is caused by astrocytes, microglia, and inflammatory mediators that cause low-grade inflammation and disruption of the blood–brain barrier (BBB) (Yin et al 2017; Zhou et al 2006). Hu and co-authors agree that inflammation can cause depression through structural remodelling of the amygdala and its functional alterations (Hu et al 2022). The findings of Yasuno et al. (2024) suggest that suppression of inflammation can help effectively treat anxiety by attenuating damage to the amygdala and its associated areas. Numerous studies clarify the reciprocal relationship between inflammation and sleep, showing inflammation is triggered by sleep loss and prolonged neuroinflammation influences sleep (Besedovsky et al 2019).

**Table 5:** studies on neuroinflammation in amygdala

References	Method	Study sample	Biochemical studies	Cognition assessment	Histological observation
Goschel et al 2023	MRI of Patients	Volume of amygdala by MRI	–	Subjective cognitive decline	Reduced volume of amygdala
León-Rodríguez et al 2022	Enzyme neuraminidase (NA), induced rats	Amygdala	↑TNF-α ↑NLRP3,	Open field test showed ↓unsupported rearing, ↑grooming & freezing	↑microglial activation
Althammer et al., 2020	Ischemic Heart failure rat model	Amygdala & hypothalamus	↑TNF- α, ↑IL-1β ↑IL-6	–	Robust microglial cell activation in central amygdala

## Feedback loops involved in neuroinflammation

Neuroinflammation in response to injury or infections in the brain, initially acute to repair the injured tissue can lead to chronic stage when sustained for a long period. It is regulated by both positive and negative feedback mechanisms or loops. Negative mechanism encourages resolution and reduces inflammation (Bhol et al 2024), while positive mechanism aggravates the inflammation (Glass et al 2010, Ofengeim et al. 2017). Understanding both the mechanisms are necessary for developing therapeutic approaches.

**Positive feedback** loop involves cytokine storms wherein initially protective pro-inflammatory cytokines trigger over-production of themself when inflammation gets sustained for a prolonged period. Simultaneously, activated microglial cells further enhance the generation of proinflammatory cytokines (Glass et al 2010) and reduces amyloid beta protein clearance through kinase RIPK1 upregulation, leading to plaque formation and apoptosis of neurons that was observed in mice model of AD (Ofengeim et al. 2017).

**Negative feedback** loop involves anti-inflammatory cytokines, neurotrophins like BDNF and transcription factors like NF-κB performaning crucial roles in the resolution of inflammation. Excessive activity of proinflammatory cytokines can be dampened by the counter activity of anti-inflammatory cytokines (Bhol et al 2024). Neurotrophic factor BDNF aids in resolving neuroinflammation by regulating the crosstalk between microglia and astrocytes. It promotes neuronal survival and encourages microglia to supervise the cyclooxygenase-2 expression and transcription factor NF-κB (Giacobbo et al 2019; Lai et al 2018; Liu et al 2017). Understanding the mechanism of both negative and positive feedback loops is advantageous while developing novel therapeutic strategies for the alleviation of neuroinflammation.

## Role of mitochondrial dysfunction and oxidative stress in neuroinflammation

Mitochondria are the major source of energy supplier for neuronal survival. Therefore, impaired mitochondrial function and resulting oxidative stress play a critical role in intensifying the process of neuroinflammation. It is reported that neuroinflammation in major neurodegenerative disorders like AD, PD or ALS are linked to impairment in mitochondrial function (Qin et al 2024; Peggion et al 2024). In AD, excessive oxidative stress impairs mitochondrial function and this vulnerability of mitochondria to oxidative stress is attributed to the lack of histones (Tan et al 2018). Removal of these impaired mitochondria by the autophagic (mitophagy) process also gets affected. Declined ATP generation contributes to the accumulation of amyloid-beta (Aβ) fibrils and hyperphosphorylated tau protein tangles, which lead to synaptic dysfunctions and cognitive impairments (Rai et al 2020; Bergamini et al 2004). In human AD brain also amyloid β accumulation was associated with mitochondrial dysfunction, as reported by Devi et al. (2006). This suggests that loss of ATP production and subsequent enhancement in ROS generation plays a pivotal role in the progression of neurodegenerative disease. The voltage-dependent anion channel proteins (VDAC) are responsible for metabolite diffusion between the mitochondria and cytosol (Hodge and Colombini 1997). Aggregated Aβ interacts with VDAC1 and blocks the mitochondrial permeability transition pore (MTP) that obstructs the diffusion of mitochondrial proteins from the nucleus. This leads to oxidative phosphorylation, enhancing free radical generation (Manczak et al 2011; Manczak and Reddy 2012). Damaged mitochondria generate damage-associated molecular patterns (DAMP) or alarmins like mtDNA, cytochrome C and cardiolipin, which can activate microglia and astrocytes and enhance production of proinflammatory cytokines by these glial cells further compromise functioning of mitochondria and escalate neuroinflammation (West et al 2011; Glass et al 2010).

## Bidirectional interaction between aggregated Aβ and hyperphosphorylated tau proteins

Aggregated Aβ and hyperphosphorylated tau proteins interact bidirectionally, to initiate neuroinflammation as well as they get accumulated along the course of the inflammatory process, further amplifying neuroinflammation. Accumulated Aβ proteins bind to the recognized receptors (TLRs) and NOD-like receptor protein 3 inflammasomes, on microglia and trigger the immune reaction by releasing the proinflammatory mediators (Islam et al 2025; Heneka et al 2015). Aggregated form of hyperphosphorylated tau proteins trigger NF-κB signaling in microglia, promoting the release of pro-inflammatory cytokines that further escalate tau pathology (Maphis et al 2015). It is suggested that the interleukin 1β that is released from activated microglia drives tau pathology. Additionally, chronic neuroinflammation augments hyperphosphorylation of tau by upregulation of GSK-3β as well as CDK5 kinases and it also hampers microglial phagocytosis and autophagy-lysosomal pathway for clearance of tau (Pascoal et al 2021; Kinney et al 2018).

## Systemic inflammation induced neuroinflammation

Recurring gut infections and cognitive impairment have long been linked to systemic inflammation (Perry et al 2003, 2007). Recent studies have revealed that peripheral insults associated with systemic inflammation when sustained for a prolonged period can lead to neuroinflammation (Perry et al 2003; Amor et al. 2014; Wendeln et al 2018; Dinan and Cryan 2017). Currently, emerging evidence implicates gut microbiota dysbiosis with several neurodegenerative disorders (Fang et al 2020). Bidirectional connection between the gut and brain, the gut-brain-axis takes place through the immune cells, vagus nerve and microbial metabolites (Collins et al 2012; Doifode et al 2020). Other than its metabolic and protective functions, gut microorganisms have a crucial role in maintaining immune homeostasis (Guarner and Malagelada 2003). A number of neuropsychiatric disorders have displayed abnormalities in the composition of gut microorganisms like autism spectrum disorder (Lim et al 2017), depression (Naseribafrouei et al 2014), AD (Cattaneo et al 2017), Parkinson’s disease (Perez-Pardo et al 2017). Therefore, modulating peripheral immune response, particularly aiming at the gut microbiota, appears to be a beneficial therapeutic approach in alleviating neuroinflammation and associated cognitive deficit (Akbari et al. 2016). These therapeutic modulators can be incorporated in the form of dietary fibres, prebiotics or probiotics, which aid in the growth of beneficial bacteria. Additionally, peripheral immune responses can be improved by short-chain fatty acids (SCFAs) metabolites, which are produced by the gut microrganisms, mainly through fermentation of dietary fibres (Silva et al 2020). They improve the T-cell responses, which help in suppressing excessive immune responses. They also promote maintenance of microglial homeostasis and thus provide an environment for reducing inflammatory response in the brain. In this way, peripheral inflammation can be modulated, and this might help indirectly in mitigating neuroinflammation and associated cognitive decline. Therefore, supplementation of SCFA either directly or indirectly through diet appears to be a promising treatment for neurodegenerative diseases.

## Animal models of neuroinflammation

There are numerous studies on different animal models of neurodegenerative diseases, to explore the neuroinflammatory mechanisms that transform acute into chronic neuroinflammation. But these models come with certain limitations, because different animal models exhibit varied pathological appearances of neurodegeneration like deposition of amyloid plaques, tau aggregation, lewy bodies or glial activation (Banik et al 2015). Major limitation is the variability in the genetic constituents of animals and humans, though the discovery of new knock-in mouse models closely mimic physiological models of AD (Drummond and Wisniewsk 2017). Though studies in non-human primates are better options because of their greater similarity in genetic combination of humans and resemblance to physiological development of AD (Rai et al. 2024, Heuer et al 2012; Jucker 2010; Teschendorf and Link 2009), there are limited studies on these models because of availability, cost and inconsistency in the extent of pathology in all animals. Hence, it is necessary to conduct preclinical testing in various animal models that closely imitate neuroinflammatory process and the complexity of the mechanism for assessing long-lasting and affordable therapeutic strategies.

## Neuroinflammation on aging and metabolic conditions

*Aging and neuroinflammation*: Aging in the healthy brain is characterized by a low-grade, chronic, and sterile inflammatory process known as neuroinflammaging (Soraci et al 2024). As age advances, reactivity of the neuroimmune environment increases owing to the elevated level of proinflammatory cytokines in response to various environmental or genetic factors. Neuroinflammaging weakens neurogenesis (Spalding et al 2013), compromises mitochondrial function (Grimm and Eckert 2017) and neuroplasticity, especially in the hippocampal areas, and in turn accelerates cognitive deterioration. Moreover, aged microglia are no longer capable of establishing effective immune responses and sustaining normal synaptic activity, directly contributing to age-associated cognitive decline and neurodegeneration (Costa et al 2021).

*Metabolic conditions and neuroinflammation*: Whereas, in metabolic conditions like diabetes and obesity systemic inflammation induces neuroinflammation, driven by adipose-derived cytokine such as leptin & IL1β, which can trigger BBB disruption and enable peripheral immune cell infiltration in the brain (De Felice and Ferreira 2014). Moreover, in diabetes, impaired insulin signaling alters PI3K/Akt and MAPK pathway, adversely affects neurogenesis, enhances oxidative stress and neuronal cell apoptosis (Arnold et al. 2018). Obesity is associated with chronic but low- grade peripheral inflammation (Zattarale et al. 2020). In obese patients, fat metabolism gets disturbed leading to augmented free fatty acid level in circulation. These fatty acids bind to pattern recognition receptors on immune cells and enhance the release of proinflammatory cytokines in the circulation triggering systemic inflammation-driven neuroinflammation (Litwiniuk et al 2021). Moreover, they also adversely affect mitochondrial function, thereby producing free radicals that increase oxidative stress (Hoeks and Schrauwen, 2012).

## Role of glymphatic system in neuroinflammation

Glial-dependent lymphatic transport, the glymphatic system eliminates waste products like metabolites, inflammatory cytokines, chemokines & soluble proteins from the brain. This function is facilitated by aquaporin-4(AQP4) water channels on astrocyte end feet, empowering exchange between CSF and interstitial fluid (Iliff et al. 2013). The glymphatic system regularly filters neurotoxic substances from the brain, primarily during sleep and in wide-awake state it is disconnected (Reddy et al. 2020; Jessen et al 2015). Sleep induces a decline in the level of noradrenaline, leading to expansion of the extracellular space, which facilitates smooth flow and filtration of the CSF, improving the waste clearance (Plog and Nedergaard 2018). Effectively functioning glymphatic system helps to maintain the microenvironment of the nervous tissue by clearing the waste and preventing build-up of neurotoxins. Weakened glymphatic system, due to various brain insults like traumatic brain injury, infections or aging can lead to reduced clearance and subsequent accumulation of inflammatory mediators (Rasmussen et al. 2018). Such dysfunctional glymphatic system is linked to the pathogenesis of neurodegenerative diseases, like AD (Reddy et al. 2020), which has the characteristic feature of amyloid β and tau protein accumulation. Consequently, dysfunctional glymphatic system intensifies neuroinflammatory process, leading to neurodegeneration induced by chronic inflammation (Plog and Nedergaard 2018). Therefore, neurotherapeutics focused on the improvement of glymphatic clearance system might help in curbing the progression of neurodegeneration (Reddy et al. 2020).

## Inflammatory cytokines on growth of the body

Though there are multiple factors that contribute to the growth of the body, it can also be affected by inflammation in early or prepubertal periods of life. Proinflammatory cytokines like TNF α plays a role in growth of the bones or tissue cell proliferation and it acts by interfering with the release of IGF 1 (Insulin like Growth Factor 1), the function of which is to manage the effects of growth hormones in the body (Zhao et al 2014; Choukair et al 2014). Due to an imbalance in the activities of these components growth of the child can be affected in an early age, leading to short stature (SS). Zinc plays a significant role in regulating inflammation through various mechanisms. It exhibits anti-inflammatory properties by effectively preventing the activation of NF-kB, a transcription factor involved in the expression of numerous proinflammatory genes (Garcia et al. 2013). Pro- inflammatory markers TNF-α and IL-6 were raised significantly in the SS group of the study (Yadav et al 2024). According to them Cu/Zn ratio along with TNF α and IL-6 can be utilized as one of the markers to investigate short stature in early years of life before puberty and they advocate Zn supplement could help in the growth of the bone by modulating TNF α and IL-6 level in chondrocytes of the developing bone.

## Therapeutic strategies

Knowing the fact that neuroinflammation is a multifaceted biological response, triggered by several factors like enhanced inflammatory cytokines, activated glial cells, gut microbiota or genetic predisposition, the development of a specific treatment approach remains a challenging procedure. Most of the therapeutic strategies for neuroinflammation-induced neurological diseases include cytokine inhibitors, microglial modulators and antioxidants. Despite the intricacy of the neuroinflammatory process and the complexity of signaling pathways of neuroimmune response, several therapeutic strategies are showing promising results (García-Dominguez 2025). These include NLRP3 inhibitors (e.g., MCC950), tetracycline derivatives (e.g. minocycline), glucocorticoids, and biologics targeting key cytokines such as IL-1β (e.g. anakinra) and TNF-α (e.g. etanercet) (Naeem et al 2024; Jiang et al. 2012; Komoltsev and Gulyaeva 2022; Mallick et al 2025) or specialized pro-resolving mediators (SPMs) like resolvins, protectins, etc. (Ponce et al 2022; Valente et al 2022; Li et al 2020). Another pathway that promotes neurogenesis, helps in maintaining BBB and thus neuroprotective is Wnt/-catenin (WβC) signaling pathway and irregularities in this pathway is associated with several well-known neurodegenerative disorders (Ramakrishna et al 2023).

### Plant based

Moreover, currently plant-derived herbal medicines are exhibiting promising results in various neurodegenerative disorder models. The bioactive compounds of several plants have shown positive results in modulating amyloid aggregation, hyperphosphorylation of tau protein, mitigating oxidative stress and neuroinflammation (Tripati et al 2024). Similarly, Indole 3 carbinol (I3C) present in cruciferous vegetables has shown the ability to inhibit Aβ aggregation by AChE modulation, in an in vitro, in silico and network pharmacology study (Ramakrishna et al 2024). According to them, these compounds can be further explored for their neuroprotective benefits since they have already shown anti-AD (Alzheimer’s Disease) through their anti-neuroinflammatory and anti-apoptotic effects in a previous study (Reyes-Hernández et al 2023). Therefore, integrating modern pharmacotherapies with such herbal medicines might effectively alleviate neurodegenerative diseases.

Likewise, in recent years research on fungal endophyte-derived bioactive compounds are exhibiting promising neuroprotective benefits and this can be mainly attributed to their antioxidant properties (Prajapati et al 2025a). Endophytes are microbes that reside within the plant tissue, developing a symbiotic relationship with the plant enhancing the growth and nutrient uptake of the host plant. Some of the endophyte-derived lead molecules have shown neuroprotective effects either by decreasing oxidative damage (Lin et al 2008; Wu et al 2015), inhibiting acetylcholine esterase activity (Vig et al 2022) or stimulating nerve growth factor, NGF (Fan et al 2023). Therefore, future studies on the discovery of novel drugs against neuroinflammation-associated diseases, can explore the genetic and metabolic capabilities of the endophytes since their bioactive compounds offer substantial neuroprotective benefits (Prajapati et al 2025a, b).

Another compound that exhibited improvement in cognition that was impaired by scopolamine, is compound 6g. Compound 6 g, a synthetic derivative of resveratrol, revealed substantial improvement in cognitive deficit in mice affected by amnesia, induced by scopolamine. This improvement in memory was associated with inhibition of AChE and reversal of oxidative stress (Tripathi et al 2019). Similarly, Srivastava et al, (2019), through their molecular docking study and in vivo study in mice, observed that compound 34 displayed antioxidant potential and cognition improvement similar to Donepezil against neurodegeneration.

### Biomaterials like nanoparticles

Many times, therapeutic drugs might not pass through the BBB because of its selective permeability and because of this treatment drugs like amyloid aggregation blockers might fail to cross the BBB. To overcome such challenges in transport of therapeutic drugs across the BBB to reach the affected region of the brain, number of research are focusing on this task. Among them, biomaterial-based drug delivery methods propose a promising result (Singh and Nagdev 2024). Biomaterials include some metals like titanium, polymers like silicon and nanoparticles like gold or silver (Gilmore and Carson 2015). They are biodegradable and compatible with the biological system, since they mimic the extracellular matrix (Ramakrishna et al 2025). Several studies recommend nanoparticle-mediated treatment strategies, since nanoparticles like gold, silver etc. have exhibited positive results in preventing neurodegeneration by facilitating transport of drugs across the BBB (Yin et al 2016; Lin et al 2016). Therefore, encapsulating the treatment drug with nanoparticles like gold facilitate delivery of drugs across the BBB (Chaturvedi et al 2021). However, commercialization and usage of biomaterials have major limitations like cost, ethical approval, accessibility etc. In addition, development of innovative biomaterials and their utilization depends heavily on intellectual property rights (IPR) which one has to struggle to obtain in this competitive world (Prajapati et al 2025b). The development, defence, along with commercialization of neuroprotective biomaterials—which show promise in treating neurodegenerative illnesses and nervous system injuries—depend heavily on intellectual property rights (IPRs).

#### Non-pharmacological approaches

In addition to pharmacotherapeutic drugs, several non-conventional therapies like exercises, dietary interventions, cognitive training etc. have shown their effectiveness in reducing neuroinflammation and improving cognitive functions. Studies have shown that the neuroinflammatory process can be modulated by regular aerobic exercises up to a certain extent (Cotman et al 2007). These exercises are known for decreasing the level of pro-inflammatory cytokines like IL-6 and TNFα, while boosting the production of neurotrophic factors. In addition, it facilitates glymphatic clearance and helps in restricting microglial activation. Other than conventional medicines, diets rich in omega-3 fatty acids and antioxidants are well known for their ability to lower inflammatory markers expression as well as to minimize oxidative stress, thereby helping in maintaining the integrity of neurons (Scarmeas et al 2018). Incorporating dietary fibres, prebiotics and probiotics that aid the growth of good bacteria in the gut also helps in the fight against neuroinflammation and associated cognitive dysfunction, by mitigating systemic inflammation (Akbari et al. 2016). Incorporating a healthy diet in the form of increased intake of omega-3-fatty acids, antioxidant rich food with a simultaneous decrease in saturated food and processed food intake. Because such healthy foods are recognized for their ability to alleviate the risk of neurodegeneration and associated cognitive decline (Pistollato et al 2018).

Cognitive training involves modifying the lifestyle by engaging in activities that enhance memory and reduce the risk of dementia (Valenzuela et al. 2008). Altogether, lifestyle interventions when applied constantly help in reducing neuroinflammation and conserving cognitive function substantially, alone or together with conventional remedies.

## Conclusion

Chronic neuroinflammation has been linked to the progression of neurodegenerative diseases like Alzheimer’s and Parkinson’s, as well as conditions like depression and schizophrenia. Understanding the neuroinflammatory responses in specific cognitive regions of the brain and the role of immune cells might provide critical insights into the mechanisms underlying cognitive impairment and neurodegeneration associated with neuroinflammation.

Though there are a significant number of treatment options for neuroinflammation and related neurodegenerative diseases, majority of them are symptomatic, because of the complexity of the mechanisms involved in the neuroinflammatory process (Gadhave et al 2024). Understanding the precise mechanism is the major challenge in the development of neuro-therapies that can effectively mitigate neuroinflammation with minimal adverse effects. In-depth knowledge about the energetic role of glial cells and peripheral immune cells in different neurological and neuropsychiatric disorders is the need of the hour in the course of discovery of the neurotherapeutics (Ransohoff 2016). Though there a number of biomarkers and novel neuroimaging techniques to monitor the progression neuroinflammatory process, still there are significant challenges that need to be addressed. Multifaceted approaches like improvement in the delivery of drugs across the BBB, disease specific cytokine centred treatment and improving the gut microbial environment with lifestyle changes would help in inhibiting the progression of neuroinflammation and associated cognitive dysfunction in various neurodegenerative diseases.This review is an attempt to summarize the reports implicating neuroinflammation in specific brain regions with cognitive deficits which would help in developing novel therapeutic strategies aiming at the modulation of neuroinflammation and improving cognitive functions.
